# Fabrication of Single-Bacterium Microgel with Gas-Shearing Strategy for Precision Probiotic Delivery in IBD Therapy

**DOI:** 10.34133/research.0955

**Published:** 2025-11-25

**Authors:** Jialin Wu, Lili Wu, Ruiying Liu, Leyan Xuan, Jiamin Qian, Chenchen Fang, Huaibin Wang, Jie Guo, Lingran Du, Yingling Miao, Bin Liu, Yutao Liu, Guosheng Tang

**Affiliations:** ^1^ Guangzhou Municipal and Guangdong Provincial Key Laboratory of Molecular Target & Clinical Pharmacology, the NMPA and State Key Laboratory of Respiratory Disease, School of Pharmaceutical Sciences and the Fifth Affiliated Hospital, Guangzhou Medical University, Guangzhou, Guangdong 511436, P. R. China.; ^2^ National Key Laboratory of Intelligent Tracking and Forecasting for Infectious Diseases, TEDA Institute of Biological Sciences and Biotechnology, Nankai University, Tianjin 300457, P. R. China.; ^3^ Division of Engineering in Medicine, Department of Medicine, Brigham and Women’s Hospital, Harvard Medical School, Cambridge, MA 02139, USA.; ^4^ School of Life Sciences, Tianjin University, Tianjin 300072, P. R. China.

## Abstract

The human gut microbiome is essential for maintaining health, as it substantially impacts immune regulation and overall balance within the body. Accordingly, disruptions in this microbial community are associated with various diseases. Probiotics offer a promising solution, but their effectiveness is often hampered by challenges related to gastrointestinal delivery. To overcome the issue of probiotic survival in the gastrointestinal system, researchers have explored various encapsulation techniques. However, traditional coarse encapsulation techniques lack precision and effective targeting, limiting the delivery of viable organisms to the colon. Current methods face challenges such as inadequate particle size control, leakage, and poor survival in complex gastrointestinal environments. This research introduces a novel approach for encapsulating individual bacteria to create single-bacterium microgels, utilizing gas-shearing technology to enhance the survival and targeting capabilities of probiotics. This approach also demonstrates the capability to coat multiple microbial species, including bacteria and fungi, while ensuring good biocompatibility and mechanical support. Focusing on *Escherichia coli* Nissle 1917, we demonstrate that this method significantly improves therapeutic efficacy in treating inflammatory bowel disease compared to unencapsulated strains. Our results suggest that gas-shearing encapsulation represents a promising strategy for the fabrication of single-bacterium microgels, facilitating the development of effective probiotic therapies with potential applications in both biomedical and nutraceutical fields.

## Introduction

The intestinal microbiota consists of a wide variety of microorganisms that inhabit the digestive systems of both humans and animals [1]. The human microbiota is primarily concentrated in the intestine, which harbors the largest and most diverse microbial population, with approximately 10 trillion microorganisms [2]. Studies on the gut microbiota have highlighted its essential roles in regulating the immune system, maintaining homeostasis, and supporting overall health [1]. Imbalances in the intestinal microbiota have been associated with a range of diseases, such as diabetes, obesity, hypertensive heart disease, inflammatory bowel disease (IBD), and certain cancers [3–6]. Therefore, modulating the gut microbiota may serve as an effective therapeutic approach to complement existing treatments for these diseases [7–9].

Oral probiotics, a class of biotherapeutics that help regulate microbial balance by producing metabolites and bioactive compounds locally, have rapidly gained recognition as a treatment for various diseases [10–12]. One major challenge is ensuring the stability of probiotics for targeted delivery to the colon when taken orally [13]. Preserving the high viability of probiotics during gastrointestinal (GI) delivery is essential for maximizing their effectiveness. They need protection from various stressors, including pH, oxygen, and temperature, throughout the stages of processing, storage, and digestion [14,15]. Preserving the high viability of probiotics during GI delivery is essential for maximizing their effectiveness [16]. Probiotic encapsulation technology is commonly employed as an efficient solution to ensure that a substantial number of viable probiotic organisms reach the colon [17–19]. At present, traditional delivery systems rely on broad encapsulation methods that group large numbers of probiotic cells together as a single entity [20]. While these multicellular encapsulation techniques provide some protection from external factors, they lack the precision and targeted effectiveness needed for optimal delivery. The rate at which the polymer matrix degrades, along with the release and absorption of the encapsulated bioactive substances in the body, is impacted by particle size. Larger particles generally release the substances more gradually over an extended period, whereas smaller particles, with a higher surface-to-volume ratio, can enhance biological adhesion and increase the efficiency of substance release [21].

Thus, the single-bacterium encapsulation technology has gained much attention. This strategy addresses issues such as inadequate particle size control, leakage, unresponsive release, low survival rates in challenging GI conditions, and reduced in vivo effectiveness [22]. Currently, research on single-bacterium encapsulation primarily focuses on the selection of encapsulation materials, while traditional methods such as stirring, shaking, and extrusion are still predominantly used for encapsulation [23]. However, the drawbacks of these traditional methods are also evident. For example, these methods often fail to provide suitable materials for all types of bacteria, as different species have unique needs regarding size, surface properties, and environmental conditions. Additionally, these methods lack the precision required to control factors like cell stability, viability, and activity. For instance, stirring or shaking can lead to cell aggregation or uneven encapsulation, compromising bacterial function and long-term stability [24,25].

**Fig. 1.**
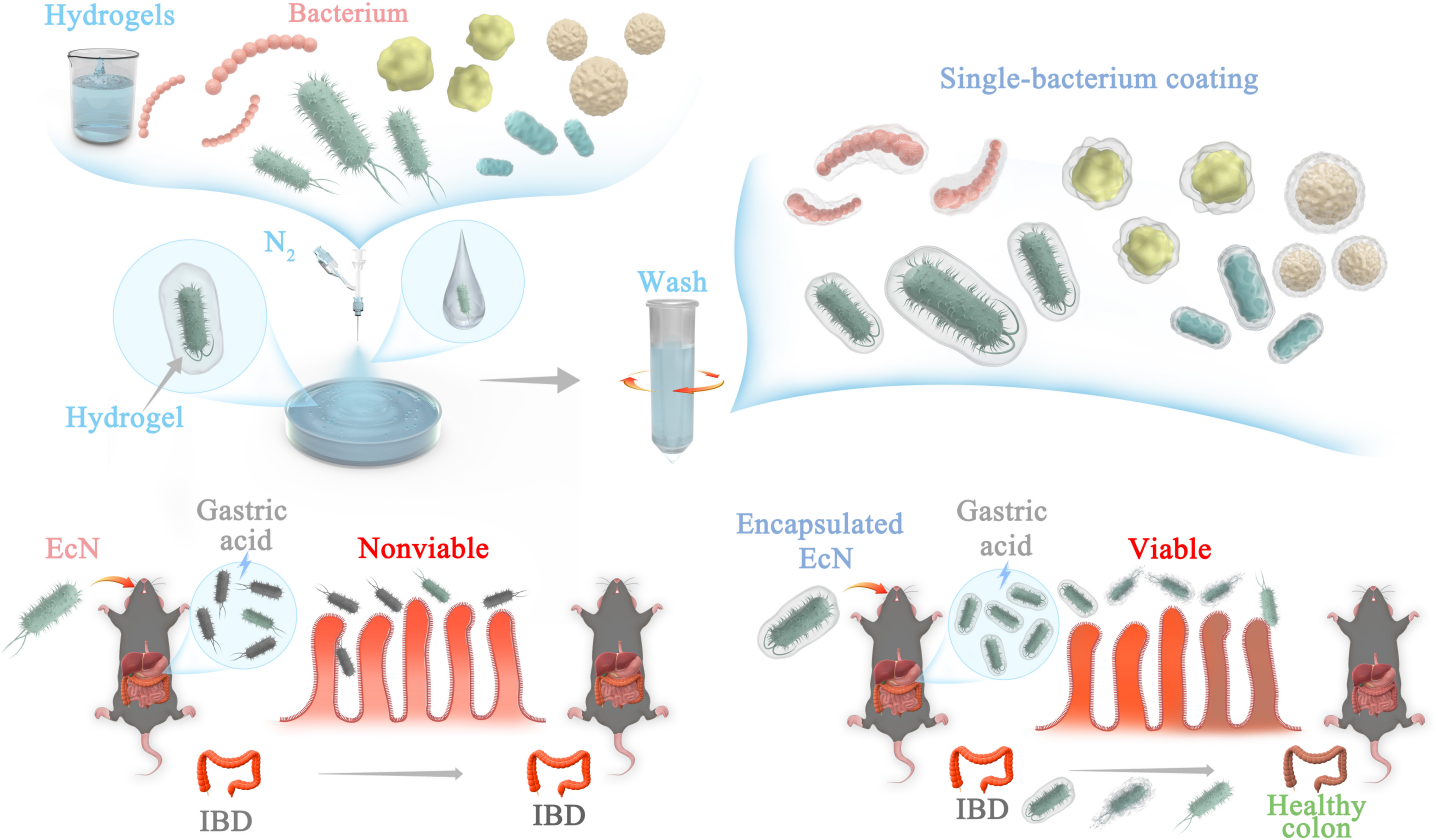
Schematic illustration of single-bacterium encapsulation using gas-shearing technology for IBD treatment.

Considering these limitations, there is a distinct necessity to innovate and advance encapsulation techniques. Inspired by our previous studies that utilize gases for fabricating microgels [26,27], we have embarked on addressing the challenges of single-bacterium microgels through gas-shearing technology. This method offers excellent biocompatibility with “soft” preparation process and without introducing aggressive chemical methods, supports large-scale production, and imposes no restrictions on the selection of coated bacteria (both Gram-negative and Gram-positive), with material selectivity. To demonstrate the benefits of our proposed single bacterial encapsulation strategy, we chose *Escherichia coli* Nissle 1917 (EcN), a gut probiotic bacterium known for its positive physiological functions and therapeutic benefits [28]. We used EcN single-bacterium microgels with this method for treating IBD, an immune-mediated intestinal disorder with complex pathophysiological mechanisms [29,30], and the efficacy was significantly improved compared to unencapsulated EcN (Fig. 1). In summary, our gas shearing-based single-bacterium microgel technique addresses the shortcomings of existing probiotic encapsulation methods and offers significant promise for diverse biomedical and nutraceutical uses involving probiotics.

## Results

### Fabrication of single-bacterium microgels

Our prior investigations involved creating soft, biocompatible multicompartmental microgels efficiently through a simple gas-shearing method, which did not require an organic phase, thus ensuring excellent biocompatibility [31]. As we demonstrated as well, microgels ranging from tens to hundreds of micrometers in size can be easily produced, with their size being precisely regulated by adjusting the flow of nitrogen gas. Based on this, we proposed that with further optimization, this approach could enable the direct creation of single-bacterium microgels as small as 10 μm or less. In gas-shearing microfluidic technology, the resulting droplet size can become infinitesimally small with the gas flow rate approaching infinity. When the gas flow rate reaches a certain threshold, individual bacteria can no longer be dispersed by the airflow, resulting in the formation of the smallest unit, namely, the single-bacterium microgels at a designed concentration of bacteria. Indeed, we optimized the process using gas-shearing spray ejector devices (SEDs), successfully achieving the encapsulation of individual bacteria (Fig. 1 and Fig. S1).

**Fig. 2.**
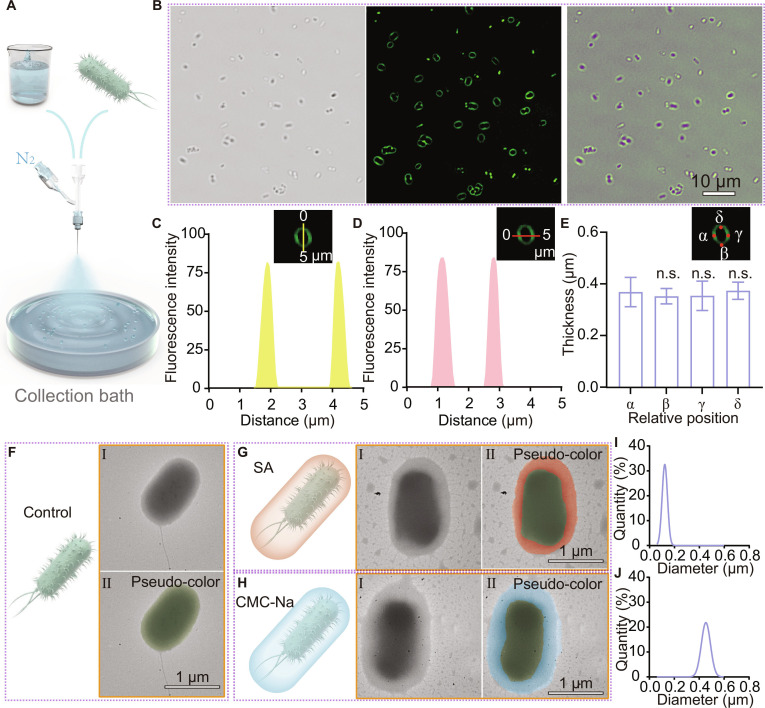
Diversity of the coating material. (A) Schematic diagram of single-bacterium microgel preparation via gas shearing. (B) Confocal images of EcN encapsulated in an SA layer mixed with fluorescent nanoparticles. From left to right, the images are bright-field channel, green fluorescence channel, and merged image. (C and D) Fluorescence intensity profiles of single-bacterium microgels. (E) Encapsulation layer thickness statistics based on relative position (*n* = 10). Significance was determined by one-way ANOVA and indicated as the *P* value; n.s., not significant; *P < 0.05, **P < 0.01, ***P < 0.001, ****P < 0.0001. Data are presented as mean ± SD. (F to H) TEM images of bacteria: the unencapsulated bacteria (F), bacteria encapsulated with SA (G), and bacteria encapsulated with CMC-Na (H). In F-H, subpanel “Ⅰ” represents the original electron microscope image while “Ⅱ” indicates the electron microscope image with pseudo-coloring applied. (I and J) Thickness statistics of EcN encapsulation with different materials, such as SA (I) and CMC-Na (J).

The gas-shearing method offers numerous advantages, including high throughput and broad material versatility. Initially, we demonstrated the microscopic wide-field imaging results of single-bacterium-encapsulating microgels fabricated via the gas-shearing technique (Fig. 2A and B). Fluorescence-based localization analysis confirmed the successful encapsulation of individual bacteria (Fig. 2C to E). To more intuitively visualize the encapsulation efficiency in this experiment, fluorescent nanoparticles were incorporated into the encapsulation layer for signal enhancement. This also highlights the customizability of the encapsulation shell, indicating that small molecules or compounds can be selectively integrated into the shell layer to meet specific application requirements. Due to the broad material versatility of the gas-shearing method, a variety of polymers can be employed as target materials for gas shearing [26]. Considering the biocompatibility and availability of the coating layer, sodium alginate (SA) and sodium carboxymethyl cellulose (CMC-Na) are taken as examples for bacterial encapsulation. Compared to the unencapsulated EcN group (control), SA and CMC-Na groups display a clearly visible complete encapsulation layer under transmission electron microscopy (TEM) (Fig. 2F to H). This indicates that the encapsulation of bacteria using the gas-shearing method allows for the selective choice of encapsulation materials. As shown in Fig. 2I and J, the thickness of the coating layer was influenced by the type of polymer, despite using the same fabrication conditions, and this could be due to variations in viscosity and/or surface tension of the 2 polymer solutions. This suggests that the thickness of the coating layer is adjustable through the selection of polymers. This collectively demonstrates that the gas-shearing method enables efficient and high-throughput encapsulation of single bacteria, with broad material versatility and customizable shell composition tailored to diverse application needs.

### Gas-shearing method for the preparation of single-bacterium microgels from multiple microbial species

**Fig. 3.**
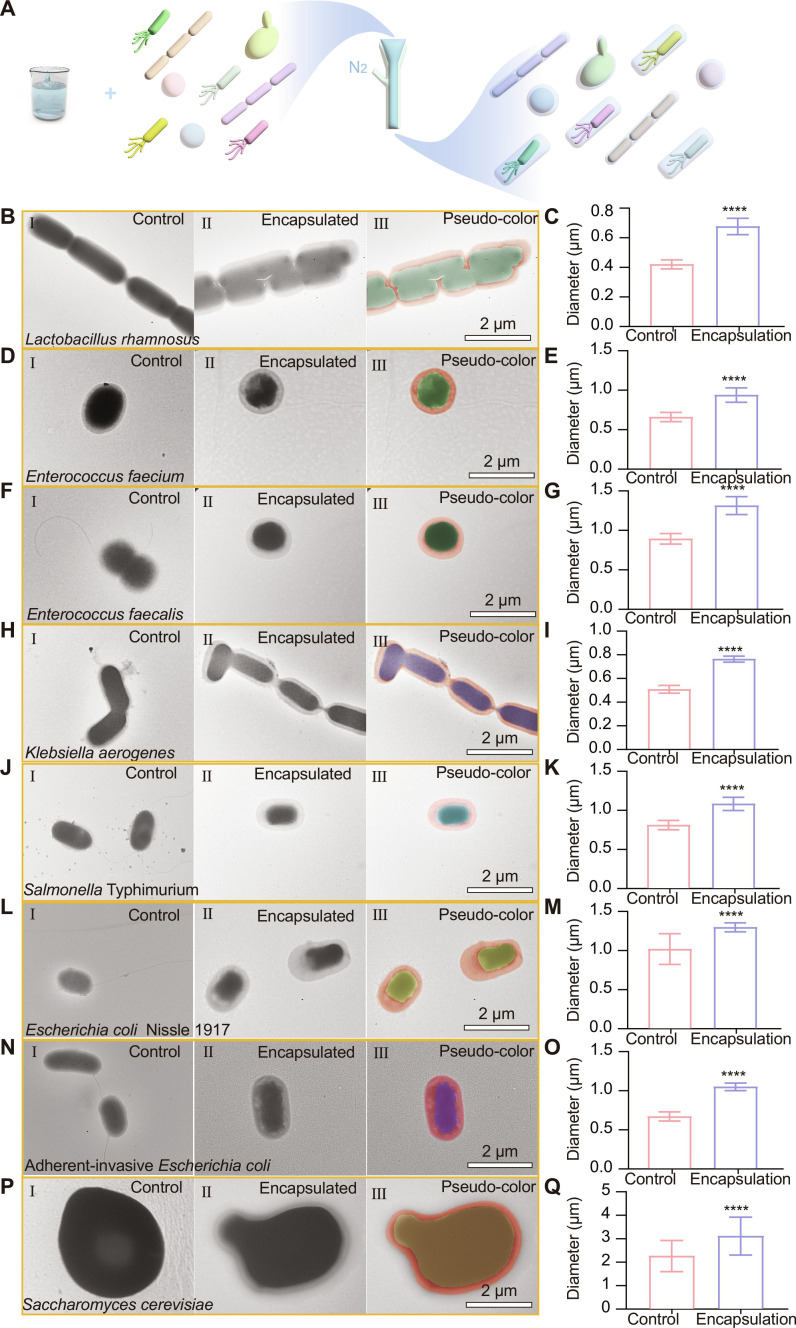
Gas-shearing method for the preparation of single-bacterium microgels from multiple microbial species. (A) Schematic diagram for the preparation of single-bacterium microgels. (B to Q) TEM images and diameter comparison of *L. rhamnosus* (B and C), *E. faecium* (D and E), *E. faecalis* (F and G), *K. aerogenes* (H and I), *Salmonella* Typhimurium (J and K), *E. coli* Nissle 1917 (L and M), adherent-invasive *E. coli* (N and O), and *S. cerevisiae* (P and Q) before and after encapsulation. I shows the unencapsulated bacteria, II displays the encapsulated bacteria, and III is a pseudo-color image of the encapsulated bacteria. Comparison chart of diameters before and after encapsulation (*n* = 100). Significance was determined by 2-tailed unpaired Student’s *t* test and indicated as the *P* value; n.s., not significant; *P < 0.05, **P < 0.01, ***P < 0.001, ****P < 0.0001. Data are presented as mean ± SD.

It is widely recognized that microbial diversity is astounding, as microorganisms exist in various forms, including fungi (such as yeast), bacteria, viruses, and algae. They play crucial roles in the human body, contributing to digestion, supporting the immune system, and maintaining overall health. The broad applicability of our technique was demonstrated by successfully encapsulating individual bacteria from different species (Fig. 3A). To prove the wide-ranging applicability of this technique, we selected *Lactobacillus rhamnosus*, *Enterococcus faecium*, *Enterococcus faecalis*, and *Klebsiella aerogenes* to represent Gram-positive bacteria, and *Salmonella* Typhimurium strain VNP20009 (VNP20009), adherent-invasive *E. coli* (AIEC), and EcN to represent Gram-negative bacteria. Additionally, *Saccharomyces cerevisiae* was chosen to represent fungi. We present the comparative TEM images of *L. rhamnosus*, *E. faecium*, *E. faecalis*, *K. aerogenes*, VNP20009, AIEC, EcN, and *S. cerevisiae* and conduct statistics on the diameters of each bacteria before and after encapsulation (Fig. 3B to Q). The results showed that the gas-shearing single-cell encapsulation technique has been demonstrated to effectively encapsulate both bacteria and *S. cerevisiae*, indicating its universality and applicability to different types of microorganisms. To further demonstrate that this method can successfully encapsulate single bacteria, we conducted mechanical testing. The analysis showed that, as opposed to the control group, the Young’s modulus of the encapsulated bacteria increased, indicating that the encapsulation layer provides better mechanical support and enhances the stability of the bacteria (Fig. S2). In conclusion, gas-shearing technology effectively and stably encapsulates single cells of various microorganisms (including bacteria and fungi) with good biocompatibility and mechanical support, making it suitable for large-scale production.

### Encapsulation of single bacterium improving the stress resistance of probiotics

Due to the pressure from the GI environment during probiotic delivery, most of the probiotics lose their activity, with only a few reaching the target site. Therefore, we conducted stress resistance tests on the probiotics before and after encapsulation to validate the effectiveness of our encapsulation strategy.

**Fig. 4.**
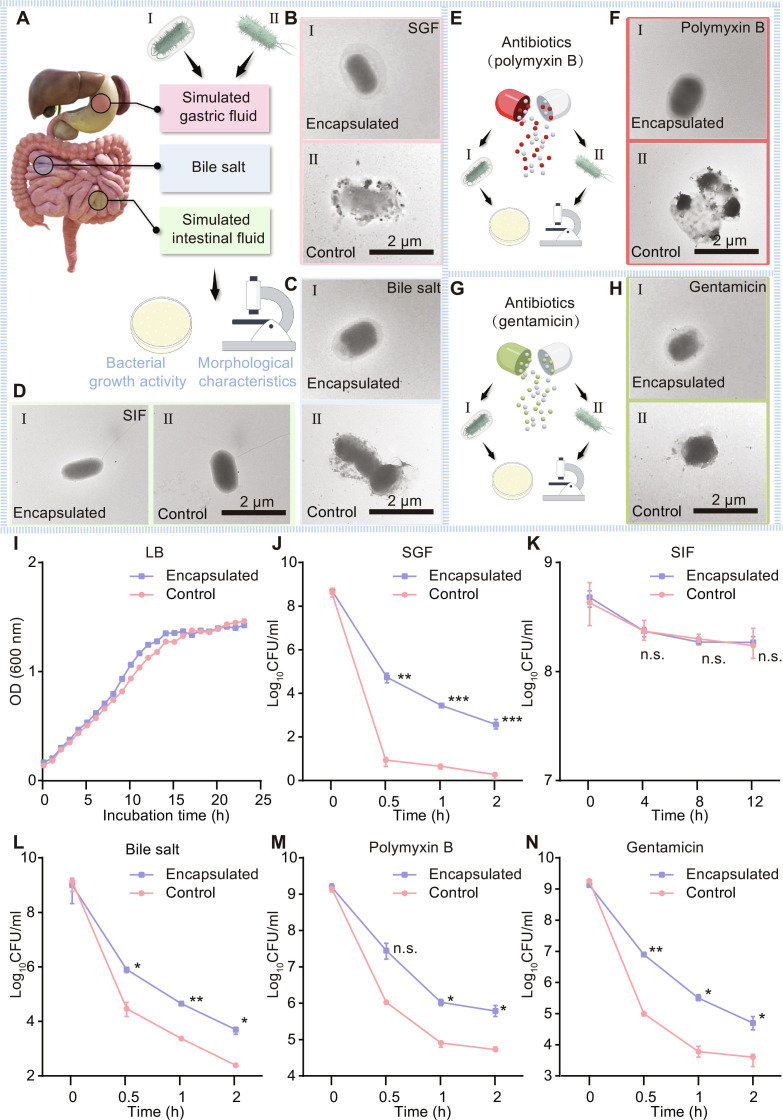
Encapsulation of single bacteria improving the stress resistance of probiotics. (A to H) Typical TEM images of en-EcN (I) and EcN (II) in SGF (B), bile salt (C), polymyxin B (F), and gentamicin (H) at 37 °C for 2 h and in SIF (D) at 37 °C for 12 h, with schematics (A, E, and G). A drop of bacterial solution was deposited onto a carbon-coated copper grid. The sample was further rinsed with double-distilled H_2_O twice and subsequently dried completely in air before observation. (I) Growth curves of EcN and en-EcN cultured in LB medium at 37 °C. (J to N) Equal amounts of uncoated EcN and en-EcN were exposed to SGF (J), SIF (K), bile salt (L), polymyxin B (M), and gentamicin (N). After the indicated time points, 100 μl of each sample was washed twice with fresh LB, spread onto LB agar plates, and incubated at 37 °C for 24 h before bacterial counting. Error bars represent standard deviation (*n* = 3). Significance was determined by a 2-tailed unpaired Student’s *t* test and indicated as the *P* value; n.s., not significant, *P < 0.05, **P < 0.01, ***P < 0.001 (J to N). Data are presented as mean ± SD (I to N).

To verify that the observed increase in resistance was due to the encapsulation layer, TEM was employed to analyze bacterial morphology after exposure to these challenging conditions. Here, we used EcN as a representative strain for the assessment. EcN, a probiotic with a well-established track record of safe use in human health [30], has demonstrated considerable therapeutic effectiveness in the treatment of cancer and GI disorders across various clinical trials [32–34]. As illustrated in Fig. 4A to H, the encapsulation layer remained intact after 2 h of exposure to simulated gastric fluid (SGF), whereas the unencapsulated EcN showed signs of damage (Fig. 4B). Similarly, the encapsulation layer successfully preserved bacterial integrity after 2 h of exposure to bile salts and antibiotics (Fig. 4C, F, and H). After 24 h of incubation in SIF, TEM showed that the encapsulation layer had dissolved, and the encapsulated EcN (en-EcN) exhibited bacterial morphology identical to that of the control group (Fig. 4D).

Next, we assessed whether encapsulation would affect bacterial growth by conducting growth curve experiments. The results showed that there were no significant differences in the growth rate during the logarithmic phase and the maximum cell density between the probiotics before and after encapsulation, indicating that the encapsulation process neither interferes with bacterial growth nor compromises bacterial viability (Fig. 4I). Building on this, we then aimed to validate whether our gas-shearing single-bacterium encapsulation strategy could safeguard probiotics from the detrimental impacts of GI conditions and antibiotic exposure.

We conducted in vitro simulation studies to evaluate the resistance of the encapsulated bacteria to GI conditions and antibiotic stress. The results are shown in Fig. 4J to N and Fig. S3. In SGF, only a few colonies of unencapsulated EcN survived after 0.5 h, whereas en-EcN retained over 100 viable colonies even after 2 h. Additionally, the bacterial density in the encapsulated group declined at a slower rate compared to the control group, and the final density of bacteria density exceeded that of the control (Fig. 4J). Additionally, we conducted similar experiments in simulated intestinal fluid (SIF), bile salts, and antibiotics (polymyxin B and gentamicin). In SIF, due to the pH responsiveness of SA [35], the 2 groups did not show any notable differences (Fig. 4K). However, results with bile salts, polymyxin B, and gentamicin were similar to those observed in SGF (Fig. 4L to N), indicating that encapsulation enhances the resilience of EcN to external conditions.

Taken together with previous results, our findings demonstrate that encapsulation enhances the stress resistance of probiotics without affecting their growth.

### Single-bacterium microgels significantly extended the residence time and increased the bacterial load in vivo

**Fig. 5.**
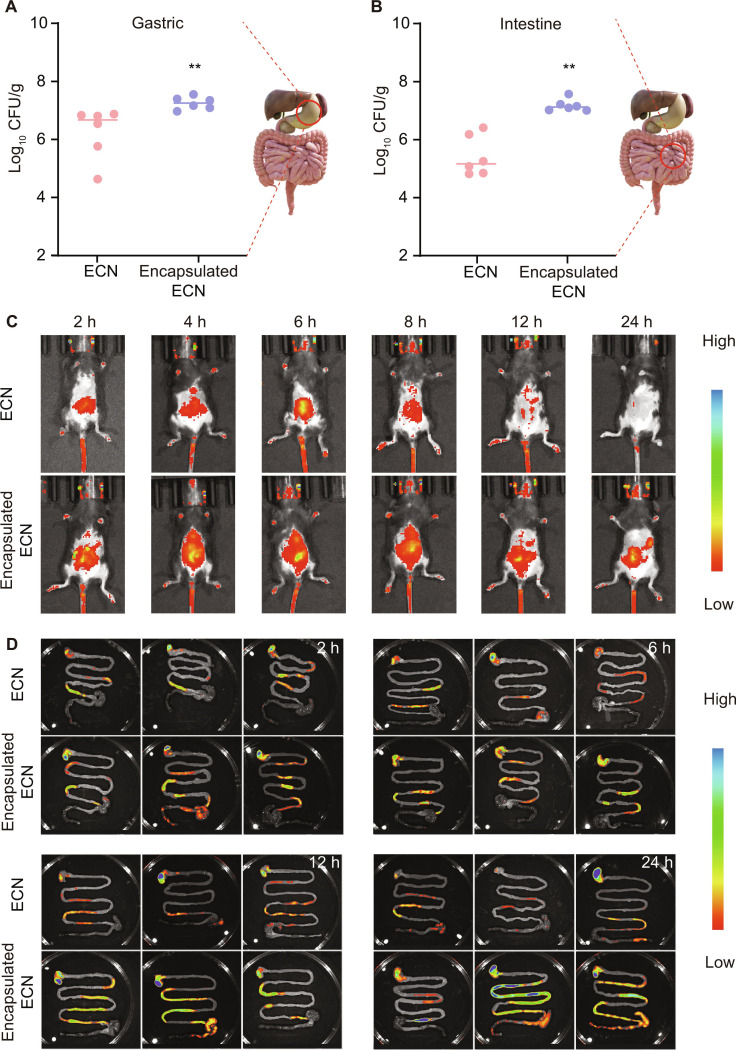
Single-bacterium microgels significantly extended the residence time and increased the bacterial load in vivo. (A and B) Evaluation of the fecal bacterial load of EcN and en-EcN in the gastric (A, collected at 2 h) and small intestine (B, collected at 6 h) of C57BL/6 (*n* = 6). (C) IVIS images of healthy mice at 2, 4, 6, 8, 12, and 24 h after oral gavage of EcN and en-EcN. Each mouse was fed with 1 × 10^8^ CFU of living EcN or en-EcN carrying pET28a-mCherry (Amp+) by gavage. (D) Ex vivo survival of EcN and en-EcN retained in the GI tract of healthy mice at 2, 6, 12, and 24 h was detected by IVIS (*n* = 3). Significance was determined by a 2-sided Mann–Whitney *U* test (A and B) and indicated as the *P* value; n.s., not significant; *P < 0.05, **P < 0.01, ***P < 0.001.

To investigate the survival benefit of EcN encapsulated in single-bacterium microgels in vivo, we compared the survival of the en-EcN with that of the unencapsulated counterpart in both the stomach and intestines. The en-EcN demonstrated a significantly improved survival rate in the stomach compared to the unencapsulated counterpart (Fig. 5A), and this advantage was even more pronounced in the small intestine (Fig. 5B). Meanwhile, we evaluated the in vivo retention time of en-EcN and the control group in mice using an in vivo imaging system (IVIS). Mice were administrated with EcN or en-EcN [1 × 10^8^ colony-forming units (CFU)] by oral gavage. There was a significant decline in fluorescence intensity in the control group mice after 12 h, whereas strong fluorescence could still be detected in the encapsulated group mice at 24 h (Fig. 5C and Fig. S4A). Encapsulation was shown to considerably prolong the duration that probiotics remain in vivo. Building upon this, we further conducted an in vivo assessment of the bacterial load. We selected 4 time points for comparison of the fluorescence intensity in the GI tract among the 2 groups. The data demonstrated that the encapsulated group exhibited higher fluorescence intensity and prolonged fluorescence retention in the GI tract compared to the control group. This suggests that the encapsulated probiotics have a longer residence time and enhanced retention in the body (Fig. 5D and Fig. S4B). To further assess long-term in vivo safety, major organs were collected from mice after 14 d of continuous administration and subjected to hematoxylin and eosin (H&E) staining (Fig. S5). No significant histopathological differences were observed between groups, indicating that the encapsulation strategy exhibits good in vivo safety. The above results indicate that single-bacterium microgels enhance the resistance of bacteria to the GI environment, leading to a longer residence time and a higher bacterial load, and exhibit good in vivo safety.

### Single-bacterium microgels of EcN showed better therapeutic effect in the murine model of colitis induced by DSS

In the modern era, IBD is recognized as a global disease, consisting of disorders that provoke chronic inflammation in the colon and small intestine, possibly resulting in more severe and fatal illnesses like colorectal cancer [36,37]. Here, we chose to use the en-EcN strain for the treatment of IBD to further explore the advantages of our single-cell encapsulation strategy.

**Fig. 6.**
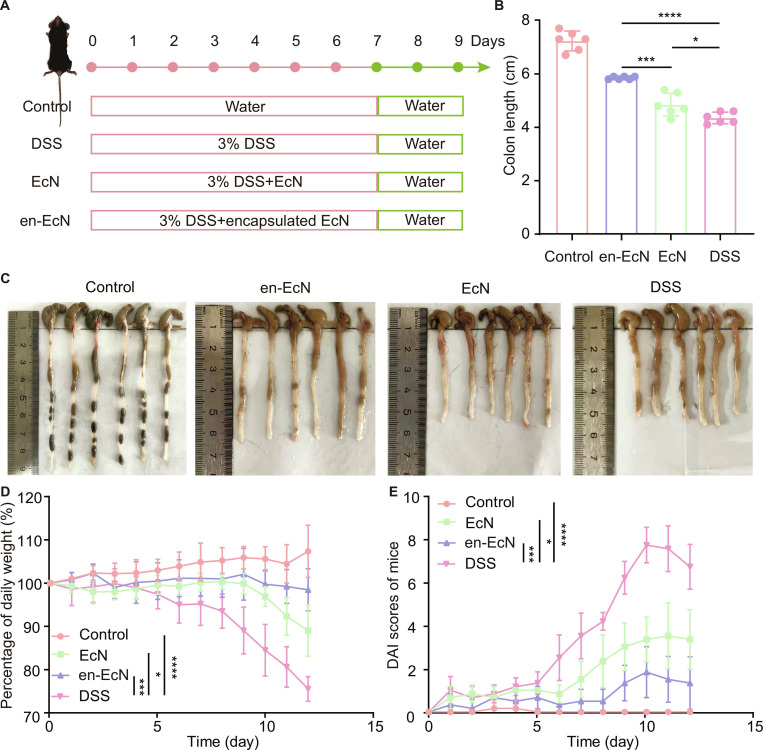
Therapeutic efficacy of en-EcN in a mouse model with DSS-induced acute colitis. (A) Schematic illustration of model building and intervention. (B) Colon length was measured and analyzed (*n* = 6). (C) Macroscopic colon appearance of each group was shown (*n* = 6). (D) Daily changes of body weight were recorded in detail and analyzed (*n* = 6). (E) Everyday disease activity index (DAI) scores were calculated and analyzed (*n* = 6). Significance was determined by a 2-tailed unpaired Student’s *t* test (B) and one-way ANOVA (D and E) and indicated as the *P* value; n.s., not significant; *P < 0.05, **P < 0.01, ***P < 0.001, ****P < 0.0001. Data are presented as mean ± SD (B, D, and E).

First, we continued by establishing a mouse model of colitis with dextran sulfate sodium (DSS) induction, which is one of the most widely employed animal models for IBD [38]. Administering DSS orally through drinking water can harm epithelial cells, compromising the intestinal barrier and leading to the invasion of luminal microbiota, which causes inflammation [39]. The experimental protocol was presented in Fig. 6A. Mice were randomly assigned to 4 groups: control, DSS, EcN, and en-EcN. The control group received sterile water, while the other groups were administered water containing 3% (w/v) DSS. Additional treatments were delivered daily via oral gavage.

**Fig. 7.**
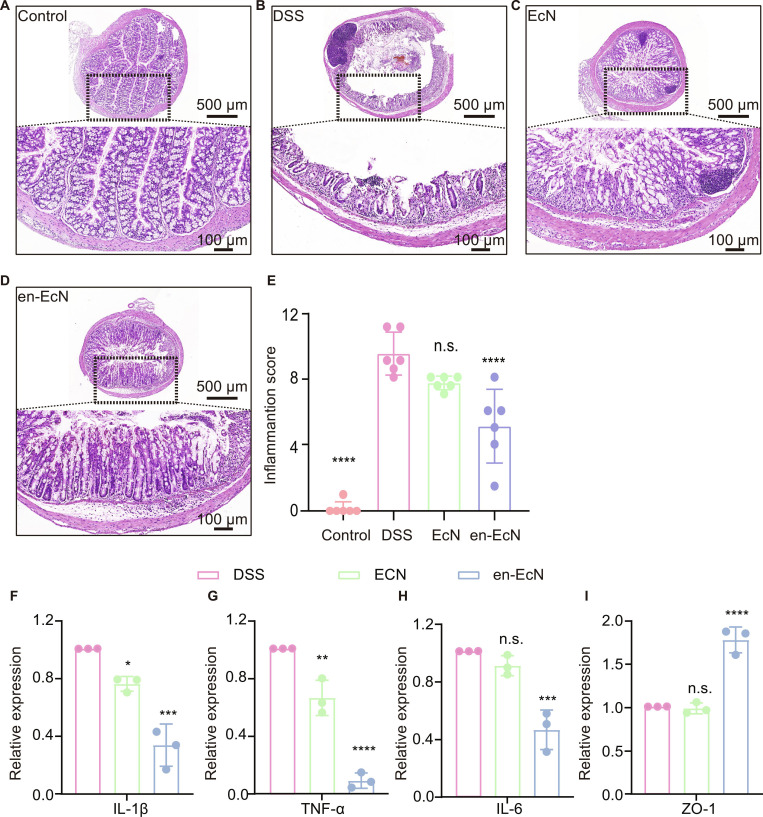
Treatment efficacy of en-EcN against DSS-induced intestinal barrier damage. (A to D) Representative hematoxylin and eosin (H&E) staining images of colon tissue of each group. Scale bar, 100 μm. (E) Colonic damage scores according to H&E staining were analyzed in each group. (F to I) Colonic mRNA levels of IL-1β, TNF-α, IL-6, and ZO-1. Significance was determined by 2-tailed unpaired Student’s *t* test (E to I) and indicated as the *P* value; n.s., not significant; *P < 0.05, **P < 0.01, ***P < 0.001, ****P < 0.0001. Data are presented as mean ± SD.

During the treatment process, we kept track of variations in the colon length, body weight, stool consistency (Fig. S6A), gut bleeding (Fig. S6B), and DAI data across 4 groups of mice. Mice treated with DSS showed reduced intestinal length (Fig. 6B and C), significant weight loss (Fig. 6D), and a high DAI score (Fig. 6E), as expected [40], while both EcN- and en-EcN-treated mice showed some degree of improvement. It is noteworthy that compared to the EcN group, the en-EcN group exhibited a lower DAI score, less weight loss, and an increase in colon length. Overall, the EcN group showed good therapeutic effects, but the en-EcN group demonstrated results closer to the control group, suggesting that encapsulation further enhanced the therapeutic efficacy of EcN. We further evaluated the efficacy of en-EcN by performing H&E staining of colon tissue. The outcomes illustrated that, when compared with the DSS group, both treatment groups exhibited increased crypt length and a reduction in plasma cells. Furthermore, the en-EcN group was more effective than the EcN group at preserving the colonic epithelium’s integrity and minimizing the infiltration of pro-inflammatory cells into the mucosa (Fig. 7A to D). Statistical analysis of the inflammation score revealed that after treatment with EcN, the inflammation score decreased, but the en-EcN group showed a more significant reduction (Fig. 7E), indicating that encapsulation further enhanced the therapeutic effect of EcN. Here, we selected interleukin-6 (IL-6), IL-1β, tumor necrosis factor-α (TNF-α), and ZO-1 as targets for analysis and compared the gene expression levels of these 4 inflammatory factors in the small intestines of mice across 4 different treatment groups. IL-6, IL-1β, and TNF-α play crucial roles in intestinal inflammation by acting as pro-inflammatory cytokines that promote the initiation and persistence of inflammatory responses [41,42]. Dysregulated expression and modulation of these cytokines may contribute to the chronicity and exacerbation of inflammatory reactions, while ZO-1 is a tight junction protein that forms tight connections between epithelial cells in the intestine, thereby regulating the permeability of the intercellular space. In patients with IBD, ZO-1 expression and function are often compromised, causing a breakdown of the intestinal mucosal barrier and allowing more bacteria and toxins to pass through [43]. Reverse transcription polymerase chain reaction (RT-PCR) experimental results showed that, according to Fig. 7F to H after treatment with EcN and en-EcN, a significant drop was observed in the expression levels of the proinflammatory genes IL-6, IL-1β, and TNF-α, the en-EcN group has a lower value, and the effect is more significant. In addition, the en-EcN group exhibited a marked increase in ZO-1 expression compared to the DSS group, while the EcN group did not significantly differ from the DSS group (Fig. 7I). The above results further confirmed that encapsulation can further enhance the therapeutic effect of EcN.

Based on the aforementioned results, compared to the EcN group, the en-EcN group demonstrated superior therapeutic effects, suggesting that our single-bacterium encapsulation strategy can effectively improve the in vivo colonization rate of probiotics, thereby enhancing their therapeutic potential in disease treatment. This provides a powerful new strategy for probiotic delivery.

## Discussion

The GI tract presents a challenging environment for probiotics, with factors such as gastric acid, bile salts, and digestive enzymes significantly reducing their survival rates. Traditional probiotic delivery systems often fail to effectively shield probiotics from these harsh conditions, resulting in reduced viability before they reach the intestinal tract. Consequently, developing more effective and precise probiotic delivery methods is crucial for improving therapeutic outcomes, particularly in treating intestinal diseases such as IBD [44–47]. While there has been considerable success in single-bacterium encapsulation technology, this strategy still faces issues such as inadequate particle size control, leakage, unresponsive release, low survival rates in challenging GI conditions, and reduced in vivo effectiveness. However, the drawbacks of these traditional methods, such as stirring, shaking, and extrusion, are also evident. For example, these methods often have poor material selectivity and lack the precision required to control factors like cell stability, viability, and activity.

In this work, we presented a simple yet robust method that took on the challenge of generating single-bacterium microgels via gas-shearing encapsulation. This method offers excellent biocompatibility with a gas-based “soft” preparation process without introducing aggressive chemical methods, supports large-scale production, and imposes no restrictions on selecting coated bacteria with material selectivity. To further demonstrate the benefits of our proposed strategy, we used EcN single-bacterium microgels for treating IBD, and the efficacy was significantly improved compared to unencapsulated EcN.

Despite the promising outcomes, this technique has some limitations that require further exploration. The degradation behavior of alginate under colonic pH conditions affects the release kinetics of probiotics. Although pH responsiveness facilitates colon-targeted delivery, variability in pH among individuals may cause inconsistent release. Additionally, the long-term storage stability of encapsulated probiotics under various conditions (e.g., lyophilization and cold chain) remains to be fully evaluated. Incorporating stabilizers or designing multilayer shells could enhance robustness during preservation and transportation. Our single-bacterium encapsulation approach offers several key advantages. From a biological perspective, encapsulating probiotics at the single-bacterium level ensures that each bacterium is fully enclosed, effectively shielding it from external threats. This complete physical barrier enhances resistance to gastric acid and bile salts, minimizes intercellular competition for nutrients, and significantly improves survival rates during GI transit. From a structural standpoint, single-bacterium encapsulation enables the formation of highly uniform microgels with excellent enclosure efficiency and low leakage risk. Such precision allows for better control of probiotic dosage and increases consistency across different batches. Moreover, gas-shearing encapsulation operates as a gentle physical process, minimizing mechanical stress on living cells and supporting high cell viability post-encapsulation. Importantly, the reduced size and uniformity of individual bacterium microgels facilitate targeted release and deeper tissue penetration while avoiding the aggregation or obstruction risks often associated with multicellular systems. This allows for precise release at specific intestinal sites, ensuring that probiotics are delivered exactly where they are needed. In our IBD mouse model, these features translated into improved colonization efficiency, stronger therapeutic responses, and better preservation of intestinal barrier function.

Functionally, the single-bacterium format also offers more flexibility for surface functionalization and environmental responsiveness. For instance, the encapsulation matrix can be engineered with stimulus-responsive elements or bioactive ligands, thereby enabling dynamic control over release kinetics, mucosal adhesion, or co-delivery of therapeutic agents. This could be particularly advantageous for combination therapies targeting complex diseases like IBD. Future work should explore material optimization and integration with synergistic strategies such as prebiotics, aiming to enhance probiotic function and gut health further.

## Materials and Methods

### Preparation and characterization of single-bacterium microgels

To achieve single-cell encapsulation, we systematically optimized the gas-shearing parameters. A nitrogen gas flow rate of 10.0 l/min and a 1% (w/v) SA (Sigma-Aldrich/9005-38-3 or CMC-Na, Sigma-Aldrich/9004-32-4) solution mixed with EcN (1 × 10^9^ CFU) were ultimately selected. The coaxial needle used in the experiments consisted of a core needle and a shell needle, with inner diameters of 0.16 and 0.84 mm, respectively. The mixed solution was continuously delivered at a flow rate of 2 ml/h using a 1-ml syringe (inner diameter, 4.5 mm) as the reservoir. The syringe was connected at its head to a flexible tube, with the other end of the tube attached to the innermost needle. A 90-mm Petri dish filled with approximately 35 ml of solution was used as the receiving bath, and the distance between the nozzle tip and the gelation bath was maintained at ~25 cm. The high gas flow rate generated sufficient shear force to disperse bacteria to the single-cell level, while the 1% alginate concentration provided appropriate viscosity for efficient droplet formation without clogging the atomization nozzle. In contrast, higher alginate concentrations, though more viscous, resulted in uneven bacterial distribution and hindered droplet formation, thereby reducing encapsulation efficiency.

Under these optimized conditions, the mixture was atomized using a coaxial needle device and dispersed into uniform droplets, which were subsequently collected in a 2% CaCl₂ (or FeCl₃) cross-linking bath. The droplets rapidly gelled upon contact with the ionic solution, forming stable single-bacterium microgels. The resulting microgels were collected and rinsed 3 times with deionized water to remove residual metal ions from the surface, yielding a clean and stable microgel system for subsequent analysis.

### Morphological and size analyses

To characterize bacterial morphology, both free and encapsulated bacteria were resuspended in water and applied to Formvar/carbon 200 mesh grids (Electron Microscopy China). After air-drying, samples were examined using a TEM (HITACHI, Japan). The size distribution of single-bacterium microgels was analyzed using ImageJ software. For confocal microscopy, 0.5% green fluorescent nanoparticles (Q-W012580, Ruixibio) were added to the SA solution, mixed thoroughly, and used to encapsulate the bacteria.

### Single-bacterium microgel viability assay

The single-bacterium microgels of EcN were incubated in Luria–Bertani medium (LB) to examine deactivation and reactivation, while unencapsulated EcN were used as a control group. Bacterial suspensions were diluted to OD_600_ = 0.2 in 100 μl of LB and incubated at 37 °C with shaking. A 200-μl aliquot was added to a 96-well plate and incubated at 37 °C, 180 rpm, for 20 h. Absorbance at 600 nm was recorded, with experiments performed in triplicate.

### Detection of the protective effect of the encapsulation layer in single-bacterium microgels on probiotics

Equal amounts of EcN and en-EcN were subjected into SGF (Coolaber SL6600), SIF (Coolaber SL6610), 0.5 mg/l polymyxin B sulfate (Sigma 5291) in phosphate-buffered saline (PBS), 4 mg/ml bile salt (Sigma 48305) in PBS, and 50 mg/l gentamicin (Sigma G3632) in PBS and held at 37 °C under shaking conditions. Bacteria were washed twice by centrifugation at 5,500 rpm for 5 min at 37 °C and resuspended in PBS. Serially diluted bacteria were plated onto LB agar and incubated for 24 h at 37 °C. Colonies were counted, with experiments repeated 3 times independently.

### Animal studies

C57BL/6 mice used in the study were acquired from the Guangdong Medical Experimental Animal Center, China. The animal study protocol, numbered GY2024-315, was sanctioned by the Animal Ethics Committee of Guangzhou Medical University, ensuring that all procedures followed the approved guidelines.

### DSS-induced colitis

To induce colitis, 3.0% (w/v) DSS (MP Biomedicals, Santa Ana, CA, USA, molecular weight 36,000 to 50,000) was dissolved in drinking water and administered for 7 d. Beginning with the first day of DSS treatment, an oral dose of 1 × 10^9^ CFU of EcN or en-EcN was administered to each mouse daily at 10 AM for 7 consecutive days.

### Investigation of the in vivo retention of single-bacterium microgels

To determine the residence time of engineered probiotics in the GI tract, female C57BL/6 mice received an oral dose of either EcN or en-EcN (1 × 10^8^ CFU). After 24 h, the mice were euthanized via cervical dislocation, and their stomachs and small intestines were removed, weighed, and homogenized using a uniform 10% dilution factor. Solid agar was used to plate a 100-μl portion of each dilution, which was then incubated for 24 h. Colonies were counted, and CFU counts were calculated using the colony numbers, dilution ratios, and plating volumes. To continue evaluating probiotic survival, female C57BL/6 mice were orally administered mCherry-ECN (EcN transformed with the pET28a-mCherry plasmid) or encapsulated mCherry-EcN (1 × 10^8^ CFU). An IVIS Imaging System (PerkinElmer, Lumina LT) in autoexposure mode was used to image the mice and their GI tracts.

### Assessment of colitis severity

During the cycles of DSS treatment, the disease activity index (DAI) scores were measured to observe the advancement of colitis. The calculation of DAI involved factors such as loss of body weight, stool texture, and the presence of blood in stool or rectum. Body weight loss was scored as follows: 0, no loss; 1, 1 to 5% loss; 2, 5 to 10% loss; 3, 10 to 20% loss; 4, >20% loss. Stool consistency was scored as follows: 0, normal; 1, soft pellets; 2, loose but solid; 3, loose and liquid; 4, watery diarrhea. Rectal bleeding was scored as follows: 0, no blood; 1, absent bleeding; 2, slight bleeding; 3, bloody diarrhea; 4, gross bleeding.

### Histological analysis

For analysis, the small intestines from infected adult mice were collected. They were fixed in 10% neutral buffered formalin overnight before being embedded in paraffin. After deparaffinization, the sections were stained with H&E and blindly scored for the severity of inflammation. The scoring for histology was based on a 0 to 3 scale for the intensity of mononuclear and polymorphonuclear infiltrates, changes in mucosal architecture, villus height, depletion of goblet cells, integrity of the epithelium, and bacterial attachment. Parameters were rated on a scale: 0 indicating absence, 1 indicating mild, 2 indicating moderate, and 3 indicating severe [48].

### RNA isolation and quantitative RT-PCR

To assess gene expression in vivo, colon samples were collected from mice. Total RNA was extracted using TRIzol Reagent (15596026; Invitrogen, Waltham, MA, USA) following the manufacturer’s instructions. RNA concentration was measured using a NanoDrop 2000 spectrophotometer (Thermo Fisher Scientific, MA, USA). cDNA synthesis was performed with the PrimeScript RT Reagent Kit with gDNA Eraser (Takara, Kusatsu, Japan). Quantitative RT-PCR was carried out on an Applied Biosystems ABI 7500 (Applied Biosystems, Waltham, MA, USA) using SYBR Green dye. The rrsA gene was used as the reference control, and relative expression levels were calculated by the 2^−ΔΔCT^ method. Each experiment was performed in triplicate.

### Statistical analyses

Our sample sizes were not predetermined by statistical methods, yet they are consistent with those reported in earlier publications [27]. The assignment of animals to control or experimental groups was random, and data were collected in line with this. The researchers were unaware of the experimental conditions. Data for other variables were collected consistently with the control group, though not randomized. All animals and data points were part of the study.

According to the figure legends, data analysis was performed using *t* tests, 2-way analysis of variance (ANOVA), or Mann–Whitney *U* tests. *P* values less than 0.05, 0.01, 0.001, or 0.0001 were regarded as statistically significant (*), highly significant (**), very highly significant (***), or extremely significant (****), respectively; n.s. denotes no significance. A normal distribution was assumed for the data, although this was not formally tested. The figures were produced using GraphPad 10.

## Data Availability

Relevant data are given within the manuscript, Supplementary Materials, and source data file.
