# Supplementary material for: Fabrication of Single-Bacterium Microgel with Gas-Shearing Strategy for Precision Probiotic Delivery in IBD Therapy
Source: Research (Wash D C). 2025 Nov 25;8:0955. doi: 10.34133/research.0955 (PMC12645448; doi:10.34133/research.0955)
Supplement: Supplementary 1 — Figs. S1 to S6 [file research.0955.f1.docx]

**Supplementary Figures**


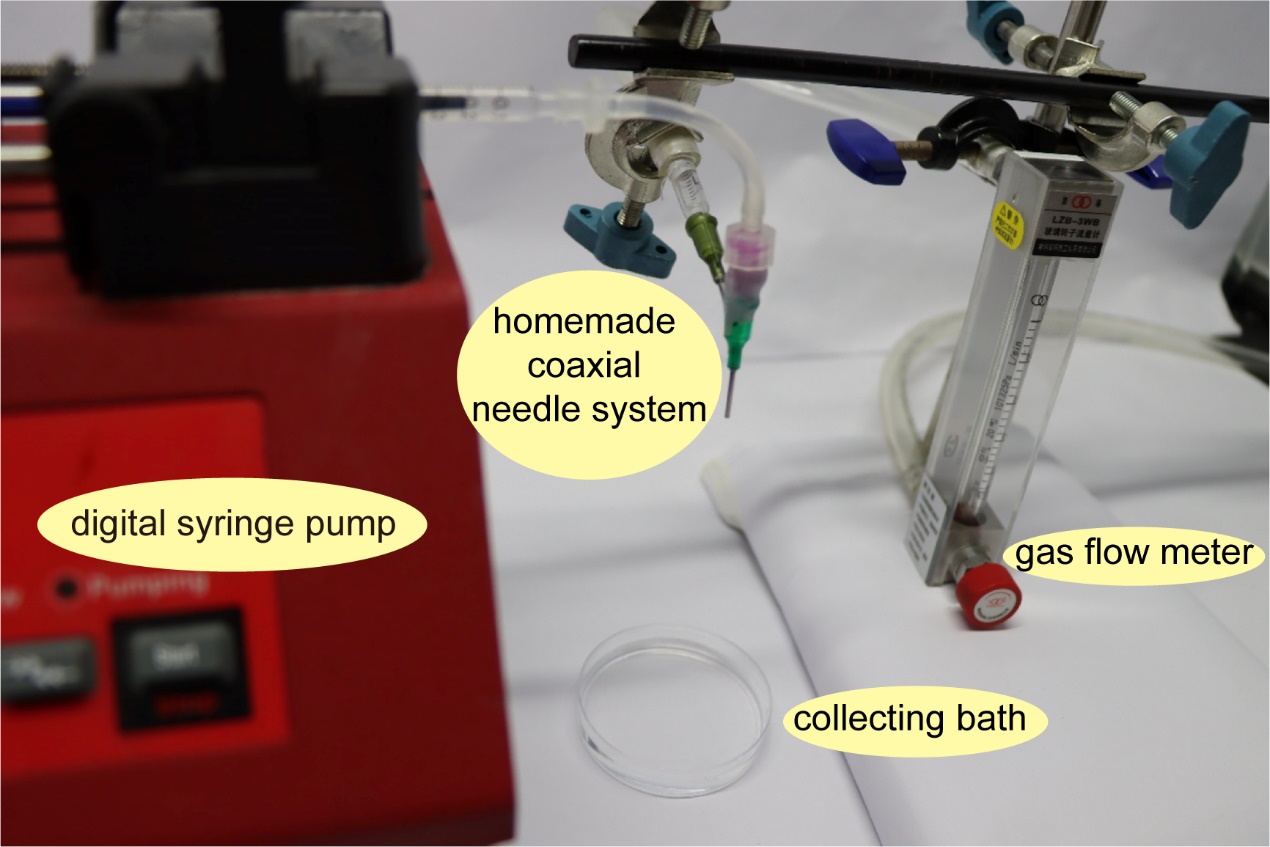


Figure S1 Photograph of a device for single-bacterium microgels using gas-shearing.


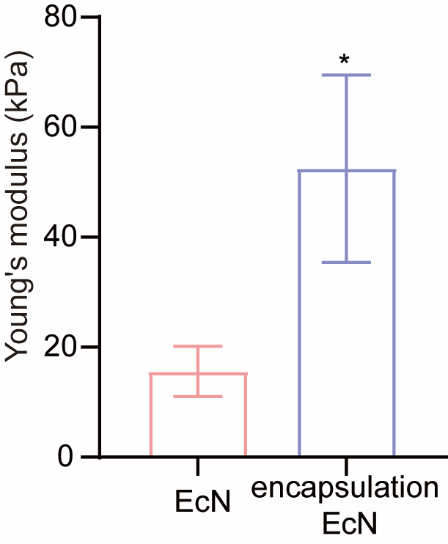


Figure S2 Young's modulus analysis of EcN and encapsulated EcN. Significance was determined by two-tailed unpaired Student’s t test and indicated as the P value; n.s., not significant; * p < 0.05, ** p < 0.01, *** p < 0.001, **** p < 0.0001. Data are presented as mean ± s.d.


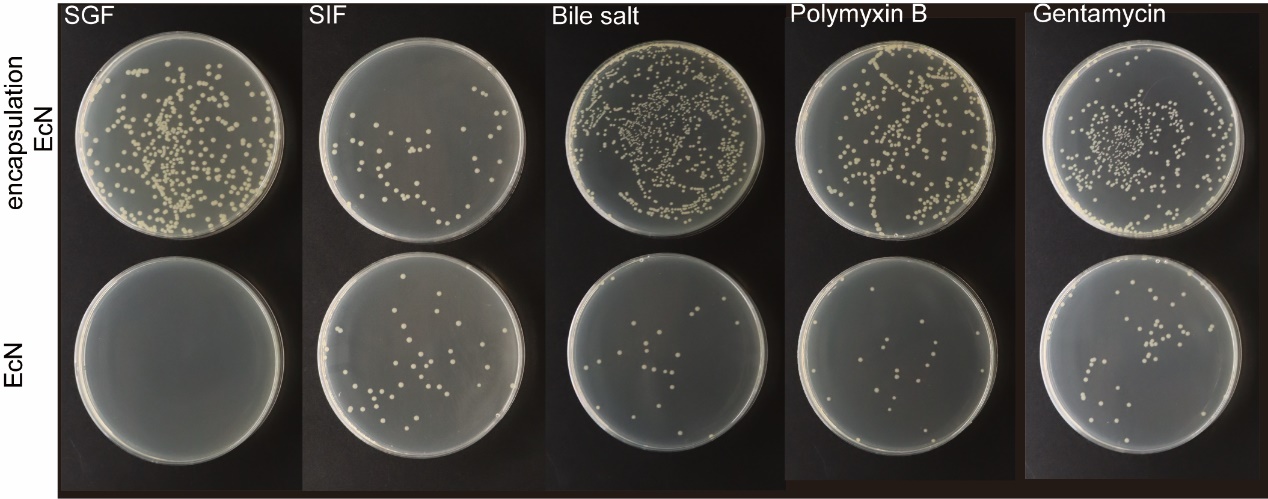


Figure S3 Representative plating images of equal amounts of EcN and encapsulated EcN exposed to SGF, SIF, bile salts, polymyxin B, and gentamicin.


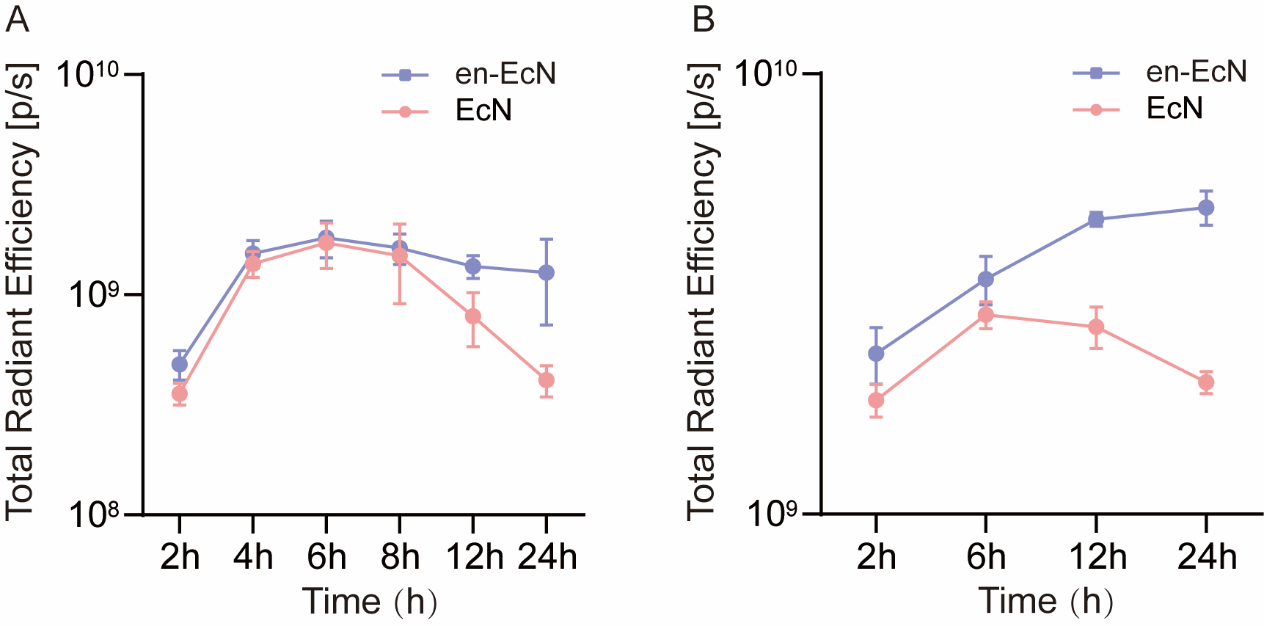


Figure S4 A, B) Quantitative analysis of IVIS imaging data (A) and ex vivo IVIS data (B). Data are presented as mean ± SD (n = 3).


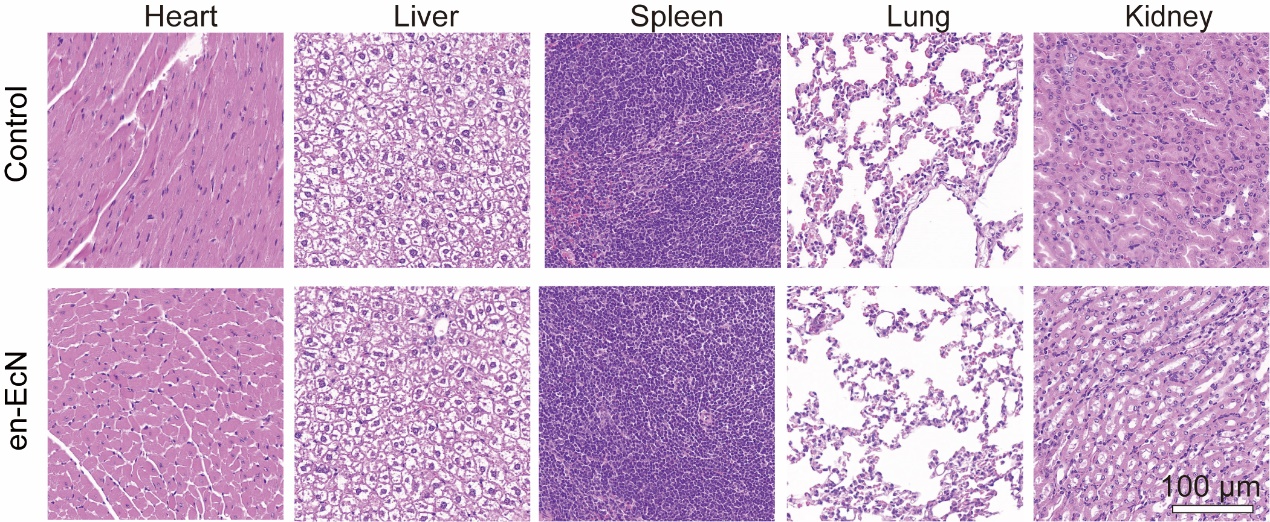


Figure S5 H&E staining of the major organ sections from C57BL/6 at day 14 under control and treatment with en-EcN.


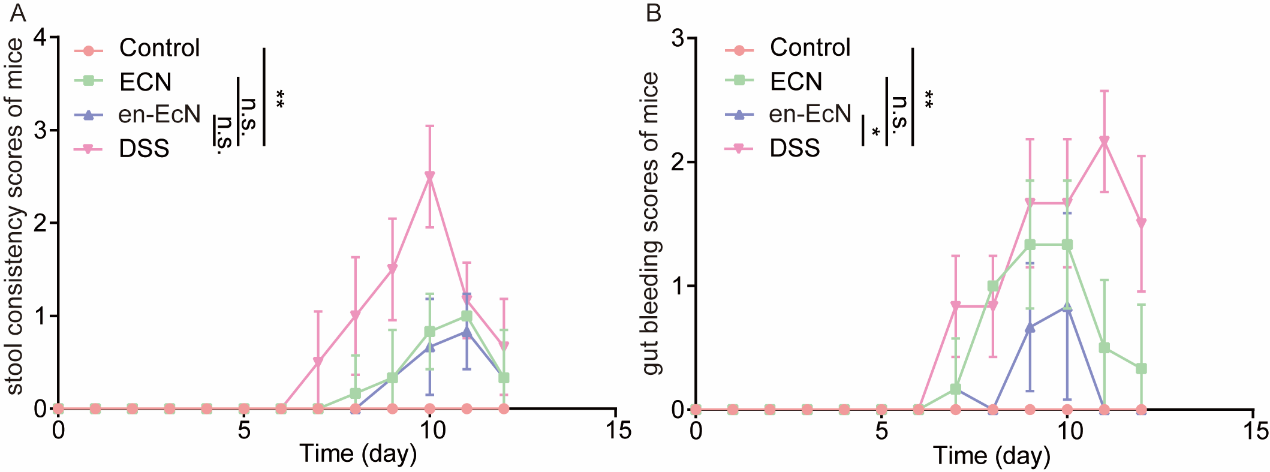


Figure S6 A, B) Stool consistency scores (A) and gut bleeding scores (B) were recorded in detail and analyzed (n=6). Significance was determined by one-way ANOVA and indicated as the P value; n.s., not significant; * p < 0.05, ** p < 0.01, *** p < 0.001, **** p < 0.0001. Data are presented as mean ± s.d.
